# What Makes Consumers Behave Sustainably When It Comes to Food Waste? An Application of the Theory of Planned Behavior in Spain

**DOI:** 10.3390/foods14132306

**Published:** 2025-06-29

**Authors:** Julieth Lizcano-Prada, Radia Ayouaz, Francisco J. Mesías, Leydis-Marcela Maestre-Matos

**Affiliations:** 1 Facultad de Ciencias Económicas y Empresariales, Universidad del Magdalena, Carrera 32, nº 22-08, Santa Marta 470004, Colombia; jlizcano@unimagdalena.edu.co (J.L.-P.); lmaestre@unimagdalena.edu.co.com (L.-M.M.-M.); 2 Escuela de Ingenierías Agrarias, Universidad de Extremadura, Avda. Adolfo Suárez, 06007 Badajoz, Spain; radia.ayouaz@gmail.com; 3 Instituto Universitario de Investigación en Recursos Agrarios, Universidad de Extremadura, Avda. Adolfo Suárez, 06007 Badajoz, Spain

**Keywords:** food waste, theory of planned behavior, cluster analysis, sustainability

## Abstract

Preventing food waste is a pressing global policy concern, with households being the main producers of food waste along the food supply chain. This study aims to analyze consumers’ food waste behavior and identify how different consumer profiles and sociodemographic characteristics influence food waste. A survey was carried out in Spain with a representative sample of 717 participants, and the Theory of Planned Behavior (TPB) was applied to understand the influence of consumers’ attitudes, subjective norms, and perceived behavior control on their intention to reduce food waste and to find out the main drivers of their food waste behaviors. Results demonstrated that food waste reduction is mainly predicted by attitudes, followed by perceived behavior control, and lastly subjective norms. Finally, characteristics such as responsibility in food purchasing and cooking at home as well as sociodemographic factors played a relevant role in how much the intention to reduce food waste affects the final behavior. Our results suggest the potential of communication to reshape individual preferences towards valuing food conservation. Tailored strategies are recommended for specific groups, emphasizing the importance of targeted approaches in addressing food waste at various levels of society.

## 1. Introduction

Nowadays, sustainability is increasingly becoming a real challenge to reduce the negative environmental impact of human activities. Within this concept, development and conservation are being combined to meet today’s needs of consumers while preserving critical ecological resources for future generations.

Food production and consumption is one of the areas where the implementation of sustainable strategies is most needed, due to the effects it has on the environment (greenhouse gas emissions, water and land use, climate change) and their foreseeable increase as the world’s population grows [1].

In this context, policies that seek to enhance the resilience of food systems are required, although their multifaceted nature implies the involvement of many actors and dimensions, such as logistics, retailing, or the loss of food that, despite being produced and transported through the chain, is not finally consumed.

Food waste, for example, is increasingly becoming a problem with a significant impact on food security, natural resources, national economies, and the environment [2]. Although figures vary according to sources, different studies illustrate the scale of the problem. According to [3], around 1.3 billion tons of food are lost or wasted globally each year—nearly one-third of all food produced for human consumption. The UNEP Food Waste Index 2021 [4] reported that approximately 931 million tons of food—equivalent to 17% of global food production—were wasted in 2019. In the European Union alone, food waste was estimated at 88 million metric tons, representing about 20% of total food production [5].

Food wastage occurs at all stages of the food supply chain [3] due to technical constraints in harvesting, storage, and cooling facilities [4] and failures in supply chain management including traceability issues and lack of infrastructure and capital [5]. Although its causes vary depending on the specific conditions and local situation in each country, food loss is mainly influenced by the type of food, the production, storage, and transport processes, and the habits or lack of awareness of consumers [6].

To properly describe this concept, various definitions and terminologies are used by different actors in the food system. In this regard, [2] emphasizes the importance of agreeing on a common definition of food loss and waste to improve information exchange as well as collaboration [2]. Therefore, in this study, FAO’s definitions are used, and hence the two terms food loss and food waste are considered as a decrease in the quantity or quality of food [7], with a small difference between them, which is mainly related to the actors involved:

- Food loss is a decrease in the quantity or quality of food that happens because of the decisions and procedures of food suppliers in the chain [7]. It occurs early in the supply chain due to a lack of infrastructure and market access [8].
- Food waste is a decrease in the quantity or quality of food where retailers, consumers, and food services are the main drivers of waste [7]. It appears further down the supply chain due to the behavior of different actors [8] as well as consumer negligence in letting food spoil or expire [2].

Reducing food loss and food waste can bring many benefits to the food system, such as cutting greenhouse gas emissions, relieving pressure on land and water resources, or increasing land productivity, thus strengthening economic growth and achieving long-term resilience [9,10]. Furthermore, a large part of the world’s population still lacks access to essential food both in quantity and quality, with one in four people suffering from moderate or severe food insecurity. Therefore, within the framework of feeding a global population expected to reach 10 billion by 2050, the fight against food waste and loss has become even more crucial [11].

This is why food waste and loss have gained relevance for the United Nations, making them appear among the sustainable development goals (SDGs number 12). The objective is to guarantee sustainable consumption and production patterns, with the aim of halving by 2030 per capita global food waste at the retail and consumer levels (SDGs 12.3) and reducing food losses along production processes and supply chains [4] by promoting 3R (reduce, reuse, recycle) practices [5].

Given the extent and complexity of food loss and waste issues, FAO has recognized the need to act in partnership with other international and regional organizations, as well as stakeholders in the food chain from farmers and fishermen to multinational retailers. Thus, the EU has joined the UN’s effort in its challenge to reduce food waste by launching the plan for the circular economy with a stakeholder platform on food waste, as well as developing methods to measure food waste and instruments to facilitate the use of these leftovers for edible surplus donation or feed production [11,12].

The approach to reducing food loss and waste is part of a broader concept to promote sustainable food systems, which takes into account, on the one hand, sustainable food production and on the other, sustainable diets and consumption (including the reduction of food waste). Measures to reduce food loss and waste should focus on systemic improvements in the efficiency and sustainability of food supply chains and should also be environmentally friendly and promote food and nutritional security [13].

Even though the reduction of food wastage is important along the food supply chain, its level of impact varies across the different stages of the chain. Thus, [14] estimates that 61% of global food waste comes from households, 26% from food service, and 13% from retail. Similarly, in the EU, households generate more than half of the total food waste (54%), with 70% of food waste arising among households, food service, and retail [15].

Hence, understanding consumer knowledge, attitudes, and behaviors related to food waste is critical to reducing food waste and improving both food sustainability and Earth stewardship [16].

Within this framework, [17] highlighted the importance of reducing household food waste as a solution to the increasing challenges of feeding the growing world’s population. Spain, as a southern European country, has distinct food consumption patterns, rooted in specific culinary traditions (Mediterranean diet) and particular social and family food dynamics. In addition, in recent years, there has been growing concern of individuals and institutions regarding sustainability and the reduction of food waste. These factors make Spain a particularly relevant scenario to explore how consumer attitudes or behavior influence household food waste practices, justifying its choice for this study.

The Theory of Planned Behavior (TPB) was chosen as the basic tool for this study because of its potential to explain consumer behavior in the context of food waste. TPB attempts to predict and understand why an individual may undertake certain behaviors [18,19]. It has been applied in different contexts and proven useful in analyzing the complexities of human social conduct [20], for example in the area of household behavior [21]. Since household food wastage is highly dependent on individual decision-making processes—such as planning, purchasing, storage, and eating habits—TPB is particularly well suited to capture the psychological and social factors that influence these actions.

Previous studies have successfully used TPB to examine food waste behaviors, [12,21,22,23] thus confirming its validity in this domain. By using the TPB, this paper provides a grounded theory approach that allows for a rigorous assessment of factors affecting food waste.

This study aims therefore to examine the factors influencing sustainable food consumption behavior in Spain, with a focus on food waste. Specifically, it investigates how the TPB constructs—attitudes, subjective norms (SN), perceived behavioral control (PBC), and intentions—affect consumer food waste behavior. Additionally, it explores the role of individuals’ involvement in food purchasing and cooking in shaping these behaviors.

This paper is structured as follows. First of all, the following section details the data collection procedure and the methodology that have been followed to carry out this research. Subsequently, in Section 3, the paper presents the main findings and discusses them based on previous studies on the topic. Finally, Section 4 outlines the main conclusions of the paper, also presenting future lines of research.

## 2. Materials and Methods

### 2.1. Data Collection

Data analyzed in this paper were collected in February–April 2023 by way of a nationwide online survey with Spanish consumers. The survey was administered by a professional market research company that was responsible for programming the questionnaire, hosting the survey, and recruiting respondents. Participants aged 18 or above were approached by email to fill out an online self-administered survey. Quota sampling according to the Spanish demographic criteria provided by the National Statistics Institute was used in terms of age and sex [24].

The research study was approved by the University of Extremadura’s Bioethics and Biosecurity Committee (registration no. 176//2022). All participants agreed to participate in the study and were assured that their answers would be kept confidential and completely anonymous. Respondents did not receive any compensation for their participation in the study.

Before implementing the questionnaire, 14 participants were chosen to pre-test the questionnaire to make sure that it was designed in an appropriate, unambiguous, and unbiased manner and that it would be valid for all possible responses [25]. This approach was also used to ensure that the questions were clear and to improve the final version of the questionnaire through minor adjustments in the wording of the questions.

The final number of valid questionnaires used in this research study was 717, following the exclusion of 62 low-quality questionnaires, which were removed due to incomplete answers. The final sample consisted mainly of women (51%) and individuals over 50 years old (42%), with individuals aged 36–50 as well (32%). These figures are similar to the demographic profile of the Spanish population [24].

### 2.2. Conceptual Framework and Data Analysis

**Figure 1:**
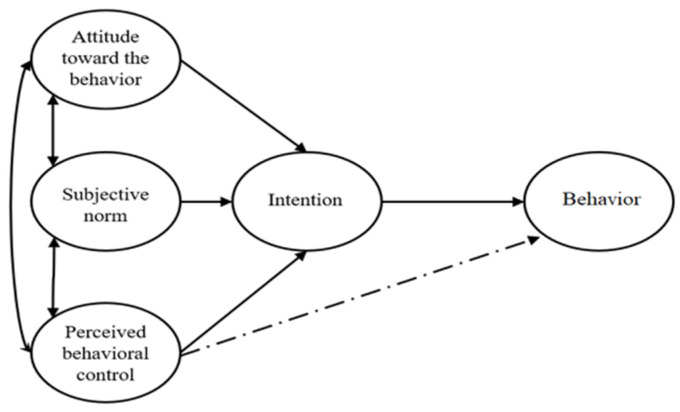
Original model of the Theory of Planned Behavior [18].

The theoretical framework used in this study is based on the Theory of Planned Behavior (TPB) [18]. TPB deals with the prediction of an individual’s behavior, which is determined by his/her intention to perform such behavior. Additionally, intention is influenced by three main determinants: attitude, SN, and PBC. Attitude is a person’s favorable or unfavorable opinions regarding the target behavior. SN is explained as a person’s perceived societal acceptability to perform or not perform the behavior [26]. Finally, PBC refers to the ease or difficulty with which the individual believes he/she can perform the behavior [27]. When these three determinants are strong, the intention will be firm, leading to the desired behavior [18], as shown in Figure 1.

TPB has been widely used to identify factors influencing sustainable food consumption behavior. Recent literature demonstrates that the core TPB variables reliably predict the intention to consume sustainable food across diverse contexts [28,29]. Taking into account the limitations of TPB—particularly its constraints in addressing emotional, cultural, contextual, and real-world factors [30]—we operationalized each TPB construct through a series of statements, including extensions aimed at enhancing the understanding of sustainable food consumption behavior (Table 1). These statements were presented to respondents, who were asked to indicate their level of agreement or disagreement using a Likert scale. Participants in the study were requested to rate them using a five-point Likert-type scale from 1 (strongly disagree) to 5 (strongly agree).

Consistent with the TPB [18], we expect attitude, SN, and PBC to account for significant variance in intention and thus emerge as positive predictors of the same. Thus, the hypotheses for this study were as follows:

**H1:**  *Attitude affects behavioral intention to reduce food waste.*

**H2:**  *SN affect behavioral intention to reduce food waste.*

**H3:**  *PBC affects behavioral intention to reduce food waste.*

**H4:**  *Intention to reduce food waste is a predictor of the final behavior.*

In order to test these hypotheses, data were analyzed using partial least squares structural equation modeling (PLS-SEM) through SMART PLS 4 software version 4.1.1.2. Structural equation modeling (SEM) is a widespread analysis technique used in different fields of social and behavioral sciences [35] to represent, estimate, and test a theoretical network of (mostly) linear relations between variables, where those variables may be either observable or directly unobservable [36].

SEM is usually based on two methods: covariance-based structural equation modeling (CB-SEM), which is based on covariance, as well as partial least squares-based structural equation modeling (PLS-SEM) which uses the variance. The PLS-SEM can be used for prediction and explanation, while CB-SEM is limited to explanation [37]. Moreover, the former provides more flexibility to explore and experiment with numerous configurations [38]. Therefore, PLS-SEM was used for this research.

The methodology applied in our analysis involves three steps.

- Description of the model: the structural model is specified, including the causal relationship between items and constructs.
- Validity and reliability of the model.
- Assessment of the structural model: to know the effect and the significance of relationships established between variables (path coefficient and *p* value).

According to [39], the coefficients used in this study are as follows: (i) standardized factor loading to test the reliability of the items to check the fulfillment of the assumption of the general linear model; (ii) Cronbach’s alpha to test the reliability of constructs among their items (internal consistency); (iii) rho_a and rho_c to test the composite reliability of constructs; (iv) Average Variance Extracted (AVE) to test the validity of constructs; (v) path coefficients to test the effect between constructs; (vi) *p*-value to test the significance of the relation between constructs.

### 2.3. Cluster Analysis

Subsequently, cluster analysis was used to allow a more in-depth study, identifying homogeneous subgroups of consumers that could reveal different behaviors regarding food waste.

Given that consumer-level waste is often due to poor meal planning and purchasing, overbuying (influenced by oversized portions and package sizes), confusion about labels (best-before and use-by dates), and inadequate storage at home, the inputs used for this segmentation were the variables “Are you in charge of household food purchasing?” and “Frequency of cooking at home” [40,41,42,43,44]. Additionally, personal involvement in cooking or food purchasing moderates the TPB-intention-behavior pathways by enhancing attitude relevance and behavioral control, strengthening value-intention links, and reducing the intention–behavior gap through practical engagement [28]. Therefore, an additional hypothesis was added to the study.

**H5:**  *Different individual profiles regarding food purchasing and cooking influence how PBC, SN, and attitude predict intention and behavior.*

Calculations were made using the cluster module of the IBM SPSS 21 statistical package, using a two-step procedure. Thus, although a hierarchical cluster is frequently used in research by [45], it was decided to use a combination of hierarchical and non-hierarchical (k-means) clustering, as various authors recommend this mixed approach, which allows the advantages of one method to compensate for the weaknesses of the other [36].

Firstly, a hierarchical clustering using Ward’s method was conducted using the abovementioned input variables. The final number of clusters was decided based on the agglomeration coefficient provided by IBM SPSS Statistics 22.0, with two solutions with 3 and 4 clusters being obtained. Subsequently, K-means cluster analyses were carried out using the cluster centroids from the hierarchical analysis as the initial cluster seeds for the non-hierarchical procedure. Finally, the criteria used to decide on the final solution were based—as recommended by [36]—on the size of the clusters obtained, the significant differences between the clusters across the clustering variables, and the external validation through the interpretation of the clusters. Taking all these into account, a three-segment solution was finally selected. A variance analysis showed that all the segments differed significantly (*p* < 0.001) from each other concerning the variables included in the analysis, which confirmed the validity of the results.

## 3. Results

### 3.1. Respondent Classification

According to the respondents’ individual purchasing and cooking frequency, this study clusters the sample into three groups. A contingency table was then made to test differences among these clusters in terms of sociodemographic characteristics. The results show significant differences, as can be observed in Table 2.

Cluster 1 (C1) predominantly consists of women heavily involved in food purchasing and cooking and is also the one with the lowest income and youngest consumers. This cluster is therefore named “Women involved in food purchasing and cooking.”

Cluster 2 (C2) is mainly made up of men and has the highest percentage of people over 55 years old. They have a middle income and actively participate in food purchasing and cooking activities. Therefore, this cluster is called “Older men quite involved in food purchasing and cooking.”

Finally, cluster 3 (C3) is primarily composed of male, middle-aged, and higher-income individuals who are in charge of daily cooking but less involved in purchasing. This cluster is named “Middle-aged affluent male foodies.”

### 3.2. SEM Analysis

Based on the theoretical model (Figure 1) and the TPB literature review, a general reflective composite measurement model was developed, in which each latent variable is measured by indicators defined in the methodology section. The SEM model was applied to the overall sample and was later replicated in each cluster following the measurement model evaluation procedure. Item reliability was assessed by considering that the loadings (λ) or simple correlations of the indicators exceeded the value λ ≥ 0.4, as indicated by [46] (Table 3).

Table 3 shows that Cronbach’s alpha coefficients [47], composite reliability (rho_a) [48], and rho_c [49] exceed the value of 0.7, indicating the reliability of the measurement models. Finally, the Average Variance Extracted (AVE) values represent at least 50% for all the constructs [50].

The discriminant validity was assessed through the [50] criterion, which compares the square root of the AVE values with the latent variable correlation. As demonstrated in Table 4, the square root value of the AVE for each construct was greater than all associated construct correlations and the discriminant validity of all scales was satisfied.

As an assessment of the model’s quality, the FIT indicator proposed by [51] was employed, yielding the standardized root mean square residual (SRMR) [52], with a value below 0.10 (Table 5) and the Normed Fit Index (NFI) > 0.90 [46].

To assess multicollinearity, variance inflation factor (VIF) values were examined. All VIF values were below the threshold of 3.3, indicating that multicollinearity is not a concern in the overall model or within the cluster-specific models. Additionally, all d_ULS and d_G values fell within the 95% percentile bootstrapped confidence intervals, supporting the model’s exact fit.

Then, the structural model results were assessed. The determination coefficient (R^2^) indicates the amount of variance in a construct explained by the predictor variables of that endogenous construct in the structural model. R^2^ values range from 0 to 1. R^2^ values should be sufficiently high to achieve a minimum level of explanatory power of this model, meeting the criteria set by [53]: a minimum R^2^ ≥ 0.10. The scale for assessing R^2^ according to [39] is: R^2^ > 0.67 is substantial; the range between 0.19 and 0.67 is moderate and R^2^ < 0.19 is weak. As can be observed in Table 5, all R^2^ values are considered moderate.

### 3.3. Hypothesis Evaluation

Table 6 summarizes the structural modeling results. These results suggest that all TPB factors significantly contribute to the reduction of food waste behavior. In the structural model evaluation, coefficients range between +1 and −1 (see Table 6: Path coefficients). It can also be noted in Table 6 that all hypotheses generate the same algebraic sign as established in the theoretical model.

The findings indicate that all TPB constructs significantly contribute to reducing food waste behavior. At the 1% significance level, all hypotheses were supported, with the exception of Hypothesis 2 for C2 “Older men quite involved in food purchasing and cooking” and C3 “Middle-aged affluent male foodies.” Consistent with the overall model, the “Attitude” construct exhibited the highest path coefficient. “PBC” presented the second-highest path coefficient, while “SN” showed the weakest effect. A strong relationship between “Intention” and “Behavior” was observed across all clusters, mirroring the pattern found in the overall model. These variations across clusters suggest that individual profile differences influence the strength of the intention–behavior relationship. Accordingly, Hypothesis 5 was supported.

Finally, the sizes of the effects of the relationships between the constructs were assessed (Table 7). The effects of Attitude → Intention and PBC → Intention were generally small, except in C3 “Middle-aged affluent male foodies,” where higher effect sizes were observed. Additionally, the effects of SN → Intention were consistently small across all clusters. The effects of Intention → Behavior were moderate, with the exception of C2 “Older men quite involved in food purchasing and cooking,” where the effect was weaker.

## 4. Discussion

### 4.1. General Model

Findings in this study related to “Attitude” being the most critical construct with a statistically significant positive correlation with “Intention” not to waste food are in line with previous studies [20,23,55].

The second most important and positive correlation with “Intention” not to waste food was “PBC.” This implies that individuals who perceived food waste prevention as an easy-to-do activity were also more likely to have a higher intention to minimize food waste. These findings challenge the conclusions of other studies that posit “PBC” as the most influential predictor of the “Intention” not to waste food [31]. Conversely, the study by [56] did not find a predictive relationship between “PBC” and the “Intention” not to waste food, although this could be explained by the age of the respondents (young people) who hence may not normally be involved in food purchasing and cooking.

Lastly, “SN” were the weakest predictor of “Behavior,” meaning that consumers’ perceived societal acceptability about wasting food affects their intention to reduce this behavior to a lesser extent than the aforementioned determinants. This contradicts the viewpoint stated in [56], which emphasizes the significant role of “SN” in predicting the “Intention” to avoid wasting food. However, this study was developed in Pakistan, a developing country where strong cultural and economic norms around food conservation and waste avoidance are shown [20].

While consumers generally express a negative “Attitude” toward food waste, their “Intention” to reduce it may be constrained by competing priorities, such as minimizing potential health risks or ensuring adequate food provision for their households. This suggests that attitude alone may not serve as a strong predictor of food waste reduction behaviour when conflicting objectives are present [57]. In the Spanish cultural context, beliefs related to food abundance and hospitality norms may inadvertently promote food waste by encouraging over-purchasing and excessive food preparation. Although many young consumers report engaging in waste-reducing behaviors, such as consuming leftovers, these practices often compete with culturally embedded habits that prioritize food abundance and are reinforced by marketing strategies and in-store promotions [58,59].

### 4.2. Cluster Comparisons

Concerning SEM at the cluster level, all clusters align with the general model, except in C1: “Women involved in food purchasing and cooking.” In this cluster, the predictability of “SN” in the “Intention” to reduce food waste outweighs that of “PBC.” This implies that individuals’ perceptions of societal expectations or norms concerning food waste reduction had a stronger impact on their intention to act than their belief in personal control over the behavior. This may be because this group was mainly composed of surveyees with lower income levels, which in previous studies were found to be more influenced by “SN” in comparison to high-income consumers [60,61].

Interestingly, C1 “Women involved in food purchasing and cooking,” with most women and young participants, is the only group with a significant relationship between “SN” and “Intention” not to waste food, contrary to expectations based on prior studies. For example, [62] observed that the effect of “SN” on the “Intention” not to waste food is stronger for men than for women, suggesting that the former rely more on the opinions and suggestions of their environment, especially from their family and friends.

In contrast, for C3 “Middle-aged affluent male foodies” and C2 “Older men quite involved in food purchasing and cooking,” the influence of “SN” on “Intention” not to waste food was not supported. This finding aligns with the notion that older and middle-aged men are less responsive to social pressure or communal expectations when it comes to food-related decisions, including efforts to reduce food waste [63]. However, other studies such as that of [64] have found that young adults reduce food waste mainly because this behavior is implanted in their social norms. These authors reported that younger consumers may be influenced to reduce food waste by their desire to maintain a positive image among their friends. This was also supported by [65], whose study showed that young adults tend to exhibit more self-control to reduce food-wasting behavior to leave a positive impression on others.

In terms of the relationship between “PBC” and “Attitude,” a stronger effect is observed in cluster 3: “Middle-aged affluent male foodies” and C2: “Older men quite involved in food purchasing and cooking,” which consist mostly of males, middle-aged, and elderly individuals with higher incomes, than in C1, “Women involved in food purchasing and cooking,” who are characterized by lower income. These findings are in line with those of [66], in which these demographics exhibit stronger proficiency in household food-related skills and express a greater sense of control over food-related activities, including the utilization of leftovers.

In terms of gender, studies conducted by [61,62] confirm that being male positively moderates the relationships between “Attitude” and “PBC” with “Intention” not to waste food. Women, on the other hand, have a higher willingness to buy items in bulk and take advantage of food discounts, which are often wasted later in households [67]. Women tend to exhibit a “good provider” behavior, focusing on caring for their family members and ensuring ample and more abundant supplies of healthy and fresh food than necessary [43]. In Spain, food waste was significantly higher among women, with plate waste most commonly involving fresh and healthy foods like bread, vegetables, and fruit, suggesting a tendency to overprovide these items [68].

Finally, and regarding age, refs. [23,61] indicate that “PBC” has a stronger effect on “Intention” not to waste food among youngsters. However, in our study, the latter is stronger among middle-aged and elderly people, probably because they were said to believe that food waste is an inevitable consequence of food consumption and therefore see it as a common and obvious practice, which does not indicate environmentally irresponsible behavior [69].

Similarly, in our study, “Attitude” has emerged as a stronger predictor among middle-aged and elderly people, in contrast with [70]’s finding that Generation Z has the largest direct association of “Attitude” with “Intention” not to waste food. The low predictive value of “Attitude” for the “Intention” not to waste food in C1, “Women involved in food purchasing and cooking,” may be explained by their income constraints and cultural beliefs regarding food, where ensuring food abundance for family members can override intentions to reduce waste [71]. However, they could also be more susceptible to over-purchasing discounted and bulk food. As a result, initial money savings from lower-priced purchases ultimately translate into higher levels of food waste later [57].

Overall, the “Behavior” is significantly influenced by the “Intention” [72]. However, according to the results of our study, this “Intention” is less predictive of the “Behavior” in C1, “Women involved in food purchasing and cooking.”

Studies such as that of [73] suggest that low-income women are more likely to prepare abundant servings because of their past experiences of scarcity, especially in bigger families. However, this could result in discarded food if leftovers are not properly managed. For these individuals, food holds symbolic significance as a representation of wealth and hope.

Our findings indicate that those with greater responsibility in food purchasing and cooking present a lower impact of “Intention” on “Behavior.” This is evidenced by C2, “Older men quite involved in food purchasing and cooking,” with lower responsibilities in purchasing and cooking, showing a greater influence of “Intention” on “Behavior,” than those in C3, “Middle-aged affluent male foodies,” and C1, “Women involved in food purchasing and cooking.”

This implies that those tasked with food purchasing and cooking might exhibit suboptimal food management practices, either during the shopping phase or when reusing food leftovers. Supporting this notion, Ref. [74] highlights that unprocessed products remain the most frequently wasted, with 73.9% of Spanish households discarding them. On the other hand, 29.5% of households waste pre-cooked dishes.

## 5. Conclusions

The analysis of food waste behavior in Spain using the Theory of Planned Behavior (TPB) confirmed the predictors—attitude, PBC, and SN—as factors affecting and explaining consumers’ food waste intention and behaviors. Factors relating to the responsibility of food purchasing and cooking allowed the classification of three consumer groups that were subsequently explained according to sociodemographic characteristics such as gender, age, and income. The findings of this study highlight the pivotal role of attitude as the most significant predictor of the intention to prevent food waste.

Moreover, this study also reflects the common observation that while intention generally predicts a reduction in food waste, the effectiveness of intention in leading to concrete behavior varies depending on the individual’s responsibility for food purchasing and preparation. This contrasts with the mixed influence of SN, which, although acknowledged in some studies as a key factor, appears less relevant in the context of competing priorities faced by individuals responsible for food purchasing and preparation.

These findings have important implications for understanding the nuanced motivations behind food waste behavior, particularly in the Spanish context, where cultural attitudes around abundance and provision further complicate efforts to reduce waste. The variation across demographic clusters, particularly the highest influence of SN among women responsible for food purchasing, points to the need for targeted interventions that consider both social pressures and practical constraints in food management.

Thus, this study contributes not only theoretically by enriching literature on TPB and food waste but also empirically, given that the insights of this research support the development of policies and campaigns to address food waste in the Spanish context. It could also help to reshape individual preferences toward valuing food conservation by communicating the importance of food waste reduction through various media, schools, populous areas, and competitions with potential awards as incentives. Tailored strategies are recommended, especially for woman and young Spanish people in charge of purchasing and cooking, low-income individuals, and younger generations.

Although the sample size presented certain limitations, the findings provide a basis for extending these results to other countries with comparable Spanish cultural and economic contexts. This study also contributes to the broader discourse on food waste by offering insights into the interplay between social norms, perceived behavioral control, and individual intentions, while underscoring the cultural and socioeconomic factors that shape these relationships. Future research should further investigate these dynamics, particularly in comparative studies emphasizing cultural, behavioral, or societal features.

## Figures and Tables

**Table 1:** Description of constructs and items.

Constructs	Items
Attitude [31,32]	
AT1	It upsets me when unused food products end up in the waste bin or garburator.
AT2	I believe that being aware about the difference between “use by” and “best before” dates is very important to reduce food waste.
AT3	Food waste is immoral while other people are starving.
AT4	I think that wasting food is a waste of money.
AT5	I sometimes think about reducing food waste.
AT6	Preventing food waste is everyone’s responsibility.
AT7	I always think about the environment when I throw away food.
Subjective Norms [33]	
SN1	Most of my family and friends are sensitive to food waste and always try to avoid it.
SN2	I do not usually provide several different types of meals so that everyone can eat what he/she likes when I have guests./I try to provide the right quantity of meals needed when I have guests to avoid leftovers.
SN3	If I generate food waste, my family/friends would find it undesirable.
SN4	In my area, social pressure makes me feel guilty when I throw away food.
SN5	Reducing household food waste will benefit future generations.
SN6	Reducing household food waste is the duty of a responsible citizen.
Perceived Behavioral Control [32]	
PBC1	I find it easy to prepare a new meal from leftovers.
PBC2	I find it easy to plan my food shopping in such a way that all the food I purchase is eaten.
PBC3	Before I prepare food, I always consider precisely how much I need to prepare and what I will do with the leftovers.
PBC4	I always plan the meals in my household ahead and I keep to this plan.
PBC5	I do not think eating food leftovers results in any health damage.
Intention [32,34]	
IN1	I intend to use all the leftovers.
IN2	I try to check the best-before dates of the food products I have at home to avoid wasting.
IN3	I intend to reduce the amount of food wasted by paying more attention to my purchases.
IN4	I intend to reduce the amount of food wasted by paying more attention to my portions.
Behavior [31,34]	
B1	I check what I have at home before food shopping.
B2	I make a shopping list before shopping and do shopping according to it.
B3	I buy the needed amount of food even when there are promotions.
B4	To minimize waste, I try to buy smaller amounts of food.
B5	In my family, the leftovers are eaten in the same form or reused in other meals.
B6	I adjust my meal plan to use leftovers.

**Table 2:** Clusters’ characteristics.

Item	Answer	C1: Women Involved in Food Purchasing and Cooking (27.9%)	C2: Older Men Quite Involved in Food Purchasing and Cooking (28.9%)	C3: Middle-Aged Affluent Male Foodies (33.2%)
In charge of household food purchasing *** (%)	Always	100	36.2	
	Sometimes		38.4	74.7
	Rarely		25.4	25.3
Frequency of cooking at home *** (%)	Daily	100		100
	Sometimes		75.6	
	Rarely		24.4	
Sex * (%)	Female	58	48.7	47.7
	Male	42	51.3	52.3
Age ** (%)	18–35 years old	31.5	26.5	22.4
	36–55 years old	32	27.2	37.1
	>55 years old	36.5	46.3	40.5
Income ** (%)	<1000 €/month	7.1	4.3	4.3
	1001–2000 €/month	29.9	30.1	18.7
	2001–3000 €/month	27.9	27.2	32.3
	>3000 €/month	35.1	38.4	44.7

Significance at: * *p* < 0.1, ** *p* < 0.05, *** *p* < 0.001.

**Table 3:** Measurement models evaluation for the overall sample and the clusters.

Construct	Indicator	Loading (λ)	Cronbach’s Alpha	Composite Reliability (rho_a)	Composite Reliability (rho_c)	Average Variance Extracted (AVE)
Overall sample						
Attitude	AT1	0.722				
	AT2	0.705				
	AT3	0.788	0.804	0.806	0.865	0.562
	AT4	0.803				
	AT6	0.725				
Behavior	B1	0.669	0.722	0.771	0.824	0.545
	B2	0.568				
	B5	0.819				
	B6	0.860				
Intention	IN1	0.652				
	IN2	0.784	0.782	0.782	0.861	0.610
	IN3	0.854				
	IN4	0.819				
PBC	PBC1	0.625	0.701	0.702	0.806	0.512
	PBC2	0.707				
	PBC3	0.774				
	PBC4	0.746				
SN	SN1	0.936	0.849	0.851	0.930	0.869
	SN2	0.928				
Women involved in food purchasing and cooking						
Attitude	AT1	0.722				
	AT2	0.724				
	AT3	0.788	0.757	0.759	0.837	0.507
	AT4	0.697				
	AT6	0.651				
Behavior	B1	0.649	0.720	0.818	0.820	0.546
	B2	0.479				
	B5	0.860				
	B6	0.891				
Intention	IN1	0.704				
	IN2	0.800	0.790	0.791	0.863	0.613
	IN3	0.838				
	IN4	0.784				
PBC	PBC1	0.680	0.704	0.713	0.816	0.526
	PBC2	0.667				
	PBC3	0.769				
	PBC4	0.780				
SN	SN1	0.952	0.890	0.893	0.948	0.901
	SN2	0.946				
Older men quite involved in food purchasing and cooking						
Attitude	AT1	0.764				
	AT2	0.739				
	AT3	0.827	0.839	0.842	0.886	0.611
	AT4	0.854				
	AT6	0.713				
Behavior	B1	0.673	0.702	0.731	0.815	0.527
	B2	0.607				
	B5	0.770				
	B6	0.832				
Intention	IN1	0.591				
	IN2	0.756	0.751	0.757	0.845	0.582
	IN3	0.562				
	IN4	0.833				
PBC	PBC1	0.656	0.701	0.703	0.779	0.504
	PBC2	0.744				
	PBC3	0.702				
	PBC4	0.635				
SN	SN1	0.935	0.832	0.842	0.922	0.856
	SN2	0.915				
Middle-aged affluent male foodies						
Attitude	AT1	0.678				
	AT2	0.656				
	AT3	0.781	0.803	0.816	0.864	0.561
	AT4	0.821				
	AT6	0.795				
Behavior	B1	0.685	0.748	0.792	0.839	0.570
	B2	0.599				
	B5	0.842				
	B6	0.862				
Intention	IN1	0.656				
	IN2	0.818	0.807	0.806	0.875	0.639
	IN3	0.870				
	IN4	0.828				
PBC	PBC1	0.545	0.702	0.715	0.808	0.585
	PBC2	0.631				
	PBC3	0.836				
	PBC4	0.804				
SN	SN1	0.924	0.834	0.834	0.923	0.858
	SN2	0.928				

**Table 4:** Discriminant validity results—Fornell Larcker criteria.

Construct	Attitudes	Behavior	Intention	PBC	SN
Overall sample					
Attitude	0.750				
Behavior	0.474	0.738			
Intention	0.505	0.492	0.781		
PBC	0.326	0.580	0.417	0.715	
SN	0.633	0.344	0.420	0.212	0.932
C1: Women involved in food purchasing and cooking					
Attitude	0.712				
Behavior	0.417	0.739			
Intention	0.484	0.440	0.783		
PBC	0.245	0.608	0.333	0.726	
SN	0.554	0.378	0.444	0.138	0.949
C2: Older men quite involved in food purchasing and cooking					
Attitude	0.781				
Behavior	0.597	0.726			
Intention	0.522	0.562	0.763		
PBC	0.382	0.570	0.433	0.685	
SN	0.674	0.419	0.425	0.252	0.925
C3: Middle-aged affluent male foodies					
Attitude	0.749				
Behavior	0.380	0.755			
Intention	0.542	0.483	0.799		
PBC	0.277	0.549	0.449	0.765	
SN	0.638	0.239	0.406	0.192	0.926

**Table 5:** Model FIT (R^2^—SRMR) ^a^.

FIT	Overall Sample	C1: Women Involved in Food Purchasing and Cooking	C2: Older Men Quite Involved in Food Purchasing and Cooking	C3: Middle-Aged Affluent Male Foodies
R^2^_Behavior	0.242	0.194	0.316	0.234
R^2^_Intention	0.343	0.328	0.346	0.396
SRMR	0.079	0.092	0.088	0.089
NFI	0.96	0.91	0.93	0.94

^a^ Categorization of R^2^ according to [39]: R^2^ < 0.19: weak; 0.19 ≤ R^2^ ≤ 0.67: moderate; R^2^ > 0.67 substantial.

**Table 6:** Hypotheses evaluation.

Variables/Tested Relationship	Path Coefficients	Standard Deviation	T Statistics	*p* Values
Overall sample				
Attitude → Intention	0.310	0.046	6.712	0.000 ***
Intention → Behavior	0.492	0.037	13.201	0.000 ***
PBC → Intention	0.281	0.036	7.819	0.000 ***
SN → Intention	0.164	0.052	3.136	0.002 **
C1: Women involved in food purchasing and cooking				
Attitude → Intention	0.289	0.092	3.148	0.002 **
Intention → Behavior	0.440	0.069	6.380	0.000 ***
PBC → Intention	0.227	0.067	3.401	0.000 ***
SN → Intention	0.253	0.103	2.451	0.014 **
C2: Older men quite involved in food purchasing and cooking				
Attitude → Intention	0.325	0.069	4.707	0.000 ***
Intention → Behavior	0.562	0.052	10.765	0.000 ***
PBC → Intention	0.274	0.059	4.642	0.000 ***
SN → Intention	0.137	0.074	1.857	0.063
C3: Middle-aged affluent male foodies				
Attitude → Intention	0.393	0.073	5.400	0.000 ***
Intention → Behavior	0.483	0.069	7.014	0.000 ***
PBC → Intention	0.322	0.055	5.900	0.000 ***
SN → Intention	0.093	0.084	1.112	0.266

Note: Asterisk indicates the level of significance of the *p*-value ** *p* < 0.05, and *** *p* < 0.01.

**Table 7:** Effect of relationships between constructs.

F-Square	Overall Sample	Effect *	C1: Women Involved in Food Purchasing and Cooking	Effect	C2: Older Men Quite Involved in Food Purchasing and Cooking	Effect	C3: Middle-Aged Affluent Male Foodies	Effect
Attitude→ Intention	0.082	Small	0.083	Small	0.080	Small	0.121	Moderate
Intention→ Behavior	0.319	Moderate	0.240	Moderate	0.463	Large	0.302	Moderate
PBC → Intention	0.108	Small	0.072	Small	0.098	Small	0.208	Moderate
SN → Intention	0.024	Small	0.066	Small	0.016	Small	0.009	Small

* Effect: Heuristic rules [54]: 0.02 ≤ f2 < 0.15: small effect; 0.15 ≤ f2 < 0.35: moderate effect; f2 ≥ 0.35: large effect.

## Data Availability

The data presented in this study are available on request from the corresponding author due to privacy and ethical restrictions.
